# Can Deep Learning Identify Early Chinese Ceramics Using Only 2D Images?

**DOI:** 10.3390/s26041312

**Published:** 2026-02-18

**Authors:** Ang Bian, Wei Wang, Andreas Nienkötter, Baofeng Di, Tian Deng, Yi Luo, Peng Chen, Xi Li

**Affiliations:** 1 School of Computer and Software Engineering, Xihua University, Chengdu 610000, China; bian@xhu.edu.cn (A.B.); chenpeng@mail.xhu.edu.cn (P.C.); lixi@mail.xhu.edu.cn (X.L.); 2 Center for Archaeological Science, Sichuan University, Chengdu 610000, China; akawwdui@gmail.com (W.W.); dibaofeng@scu.edu.cn (B.D.); 3 College of Computer Science, Sichuan University, Chengdu 610000, China; 4 Institute for Disaster Management and Reconstruction, Sichuan University-Hong Kong Polytechnic University, Chengdu 610000, China; 5 School of Electronic Information and Communications, Huazhong University of Science and Technology, Wuhan 430000, China; tiandeng@hust.edu.cn; 6 School of Architecture and Art Design, Jiangxi University of Science and Technology, Ganzhou 341000, China; luoyi@jxust.edu.cn

**Keywords:** early Chinese ceramic identification, deep learning, ceramic dating, ceramic feature recognition

## Abstract

Study of early Chinese ceramics is crucial for understanding cultural, economic, and technological developments in Chinese history. With the evolving deep learning techniques, one urgent question would be, whether we can identify early Chinese ceramics by a simple 2D image without further domain knowledge. This work collected a highly diverse dataset for ancient Chinese ceramics from 15 dynasties, with 4 representative glaze colors and 15 shape types. We studied the performance of five state-of-the-art neural networks on two identification tasks: ceramic visual feature recognition and early Chinese ceramic dating. A class-imbalance learning strategy is designed to improve the models’ performance on multi-label tasks. To the best of our knowledge, our work is the first to introduce deep learning into early Chinese ceramic recognition on a large scale. Experiments prove that deep learning can recognize visual features like glaze and most shape types with high accuracy, while ceramic dating is feasible for the main dynasties but remains challenging along the overall history. Further quantitative assessment shows that cultural inheritance and artistic continuity can lead to reasonable false dating by classifying ceramics into adjacent dynasties or periods. Moreover, although domain knowledge is required for interpretation, deep learning shows great potential in recognizing even unlabeled time-relevant features, which can help study the inheritance and evolution of early Chinese ceramic development.

## 1. Introduction

Ceramic wares, including pottery and porcelain, are important household products and precious art collections throughout Chinese history. The earliest manufacture of simple pottery can be traced back to the Neolithic Age, matured in the Han, Tang, and Song Dynasties, and peaked in the Ming and Qing Dynasties. Ceramics production has developed into numerous techniques and styles, such as the Tang Tri-Color Glazed Ceramics and the Blue-and-White porcelain of the Ming Dynasty, which are considered masterpieces of craftsmanship. Investigating early ceramics antiques helps in understanding early Chinese history in manufacturing techniques, crafting techniques, economy, and art.

Beyond identifying attributes such as color, shape, and decorative motifs, ceramic dating, which determines the dynastic origin of artifacts, is a critical prerequisite for research on early Chinese ceramics. Senior domain knowledge and experience are generally required for feature identification, and laboratory examination methods are required for ceramic dating. However, these examinations are often complicated and need expensive physical or chemical techniques such as thermoluminescent analysis, X-ray diffraction, or Raman spectroscopy. Moreover, many of these lab tests like thermoluminescent are destructive which is unwanted for valuable historical artifacts [1,2,3,4,5,6,7]. Thus, non-destructive identification methods using optical images would be an ideal alternative.

However, existing works all focus on special porcelains (like blue and white porcelains), where dedicated features are designed using distinguishable craftsmanship and motive patterns [3,8]. With the rapid development of deep learning methods and their surpassing performances in various applications, it would be interesting to know: (1) Can deep neural networks learn the patterns that are generally acknowledged by historians? (2) If so, can deep neural networks identify early Chinese ceramics using only 2D images without any domain knowledge-based feature selection?

To answer these questions, (1) a total number of 12,253 images covering early Chinese ceramic wares from the Neolithic to the Modern is collected, labeled, and organized into glaze color, shape type, and dating datasets; (2) a series of experiments are conducted to verify the performance of several deep neural networks for two typical identification tasks: color and shape recognition to validate the efficacy of deep learning for common feature recognition, and early Chinese ceramic dating, which is the key task of this study. In our work, a new training strategy is proposed to address the severe class imbalance problem present in the collected dataset. We have further analyzed the cultural inheritance and its impacts on ceramic identification.

Our paper delivers a contribution to the AI-based cultural heritage application by showing that deep learning can classify glaze colors, shapes, and dynasties of early Chinese ceramics using simple 2D museum photos. The large labeled ancient Chinese ceramic image dataset fills a real gap as a baseline for future scientometric studies on regional ceramic disparities, with comprehensive evaluation results on multiple representative neural networks. This work also serves as a baseline for handling class-imbalance, while our class-imbalance learning strategy significantly boosts the classification performance of all models, especially for shape recognition and All-Dynasty dating tasks, which include multiple severe few-shot categories.

The remainder of this paper is organized as follows. Related works on ceramics identification and deep learning techniques used in this work are introduced in Section 2. Section 3 details the data acquisition, preparation, and methodologies used in this study. Experiment results are presented in Section 4. Section 5 discusses the influence of cultural inheritance on ceramic dating and the potential of model interpretation on time-relevant feature recognition. Section 6 concludes this work.

## 2. Related Work

Ancient ceramic dating is an important part of Chinese cultural heritage research. The most common dating method is to analyze the chemical composition of the glaze on the surface of the ceramics. This helps to identify the firing techniques and development of porcelain from specific dynasties or kilns [1,2,3,4,6,7]. Other works have analyzed the raw materials of ceramics in different places to reflect the geographical characteristics of ceramic development in different regions, highlighting the geographic value of ceramic research [5]. However, the current chemical or physical dating approaches can be destructive due to the sampling operation and require expensive and complicated equipment. Even though technologies like PIXE can be used for non-destructive identification [9,10], the particularly complex process is not preferable given the huge amount of unlabeled ceramic wares for daily use.

Techniques such as image processing and machine learning have been introduced for the digital management of ancient ceramics, where most studies focus on the classification of pottery sherds from Italy or Spain [11,12,13,14,15,16]. For instance, Di Angelo and his team proposed a 3D-scanned high-point density model-based measurement method and a morphological and geometrical feature analysis method to help the recovery and restoration of ancient pottery sherds [11,12]; Barak, I., Lior, W., Nachum, D. et al. implemented a deep learning-based ArchAIDE APP to recognize the outline shape and decoration of pottery sherds [13,14]; they also proposed using VASESKETCH to reconstruct 3D pottery from 2D paper catalog drawings [15]; Navarro, P. et al. have shown that, without feature selection, ResNet18 achieved much better shape classification accuracy on Iberian wheel-made pottery vessels’ profile using binary images [16]. Outside Europe, there are methods like the thorough analysis tool called Snowvision for extracting and identifying the stamped curve patterns of North American Indigenous wooden paddles from pottery fragments [17].

China has a long and skillful history of pottery and porcelain firing, especially various well-developed glazed porcelains, which represent a remarkable cultural heritage. The development of Chinese ceramic color, shape, and other features is of fundamental importance for archaeological understanding of the corresponding historical periods. Given the rich development branches of early Chinese ceramics, most works in this field focus on a certain type or characteristic of ceramics to understand the craftsmanship or motif pattern development [4,8,17,18]. Zhao, H. et al. sampled and analyzed the spectral features of Blue-and-White porcelain samples using hyperspectral techniques, and used random forest together with long short-term memory (LSTM) neural network to identify the production period [11]. Sun, J. et al. used the SLIC-Ncut algorithm to study the shape feature of porcelain pots from the Tang, Song, and Yuan periods [18]. Chuen-Horng Lin et al. established a basis for the digital management of museums, as well as the retrieval of texture features of porcelain [19].

Computer vision methods have been vastly improved with the success of deep learning, and are widely used in industry or agriculture applications [20,21]. Besides the early success of CNN in image recognition, VGG and ResNet proved that deeper neural networks are possible and effective [22,23]. The Inception model is proposed to increase the neural network’s representation ability [24]. The Vision Transformer (ViT) has achieved great success on large-scale datasets through processing of image patches [25]. Previous works have shown that deep learning can achieve 96% accuracy on the relatively simple task for Iberian wheel-made pottery vessels’ profile recognition using binary images [16], but only maximal 30.5% and 55.2% top 1 accuracy on Terra Sigillata Italica pottery sherds’ outline shape and decoration identification [14]. Our work studies the recognition ability of deep learning on the much more complicated early Chinese ceramics with rich color, texture, and shape features on a large scale.

## 3. Materials and Methods

### 3.1. Research Material Database

To ensure the diversity of our research data, we obtained 12,253 digital images online for the same amount of early Chinese ceramic wares covering all main production periods, including labels extracted from the detailed online description. The dataset is the basis for ceramic feature recognition and dating tasks.

Our dataset comprises images all in JPEG format with diverse sizes. Specifically, the height ranges from 162 to 8502 pixels, and the width spans 188 to 8275 pixels. The average image dimensions are [595, 707], with the median at [600, 799]. Notably, 90% of the images fall within the size range of [300–900, 300–1100]. For the aspect ratio (defined as width/height), its values in the dataset range from 0.625 to 1.86, with a mean of 1.22 and a median of 1.33. All images are compressed into [224, 224, 3] or [299, 299, 3] for training, validation and testing according to the default input size of each model.

**Figure 1:**
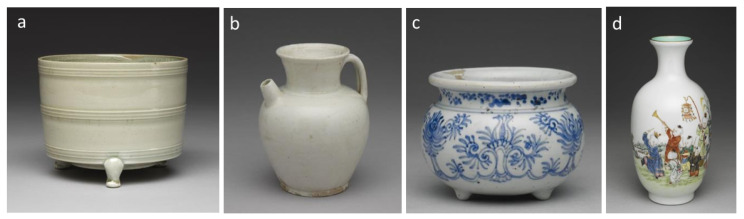
Early Chinese ceramics of 4 representative colors. From left to right are (**a**) Celadon Tripod Censer with String Patterns; (**b**) White Glaze Jug with Handler; (**c**) Blue-and-White Tripod Censer with Foreign Lotus Motifs; (**d**) Famille-Rose Vase with Boys-at-Play Motifs.

Color and shape are the two most important visual features for early Chinese ceramic identification. Our study focuses on the 4 most representative colors in Chinese ceramic firing technique development, i.e., celadon, white, underglaze, and overglaze in chronological order. The glaze color is a vital time-relevant feature for the firing skill and hence the crafting period. Celadon was created with increased firing temperature by structural improvement of kilns and predominated at least from the Han dynasty (206 BC–220). The rapid development of celadon in northern China led to the appearance of white porcelain, and both celadon and white porcelain reached their peak in the Song dynasty (960–1279). The maturation of the pattern and coloring techniques drove the prosperity of underglaze ceramics during the Ming Dynasty (1368–1644). Overglaze porcelains with multiple or bright colors like Doucai, and Famille-Rose were popular in the Qing dynasty (1616–1911). Our glaze color dataset includes 8917 images of 2464 celadon ceramics, 1733 white ceramics, 2436 underglaze, and 2284 overglaze porcelains. Figure 1 presents some early Chinese ceramics of the 4 representative colors. As can be seen, the glaze type is not that distinguishable just by the ground color; glossiness and patterns are also important.

**Figure 2:**
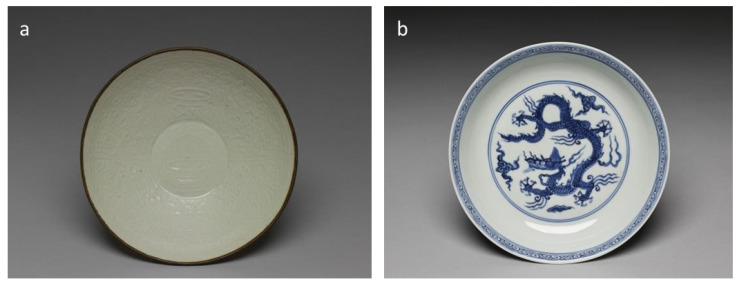
Similarity of Bowl and Plate photographed from above. (**a**) Celadon Bowl with Molded Antique Flower Patterns; (**b**) Blue-and-White Plate with Dragon Motifse.

**Figure 3:**
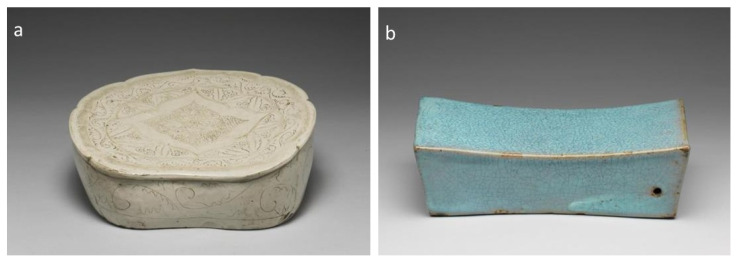
Pillows with different shapes. (**a**) White Glazed Pillow with Incised Ruyi Patterns; (**b**) Sky-Blue Glazed Rectangular Pillow.

Ceramics are widely used for all kinds of daily uses, and their shape type is another important visual feature for early Chinese ceramic identification. Chinese ceramics have developed into diversified shapes for similar functions in various scenarios or social classes. For instance, Bottles, Pots, Jugs, and Crocks can all be used for water containers, but are of different sizes to be used outdoors or indoors. Many subtypes are also developed for more specific usage or styles. For example, a cup can be designed with or without handles or a lid, with a narrow or wide mouth, for alcohol, water, or tea drinking. Ceramic wares are also found in ancient Chinese sacrificial ceremonies and funeral culture, where special shapes like Figure (俑) and Zun (尊) are found in archaeological investigations. With the ceramic image dataset, we extracted the shape labels from the online description, removed the subtypes with under 15 samples and pieces missing shape information, and finally reorganized the 23 subclasses into 15 main classes by their similarity in usage and shape. Thus, a shape type recognition dataset consisting of 10,786 ceramic wares with 15 main shape labels is built. Note that some shape types are not that distinguishable by 2D images, as shown in Figure 2, both Bowl and Plate photographed from above are of similar circle shapes. Meanwhile, special shape types like Pillows in Figure 3, can be of very different designs. Table 1 and Table 2 demonstrate the details of the two feature recognition datasets.

Evidence shows that the early Chinese pottery relics can be traced back to the Neolithic (6000 BC–2000 BC), and the history of potteries and later porcelains as one of the most common daily necessities developed along with all the Chinese historical periods and dynasties. In our collected dataset, ceramics are from all 16 dynasties or periods, and images with unknown dynasty information are neglected. Given the short history of the Sui dynasty, we take the label “Sui Tang” for ceramics from Sui and Tang, the two proximate dynasties. The Qin dynasty is excluded as only 1 ceramic piece is collected. Thus, an All-Dynasty ceramic dating dataset of 12,223 ceramic wares with 15 period/dynasty labels is built. All the corresponding period/dynasty labels are extracted from online descriptions. The overview of the All-Dynasty dataset in Table 3 shows the number of collected ceramics in each dynasty or period. Note that most ceramic relics are discovered from the 4 most thriving and prosperous dynasties, i.e., Sui Tang, Song, Ming, and Qing, showing that ceramics manufacturing benefited from the peaceful and stable economic environment.

Given the difficulty of the early Chinese ceramic dating task, we further investigate on a subset named the Main-Dynasty dataset. It consists of the major ceramic collections from the 4 main periods or dynasties: Sui Tang, Song, Ming, and Qing, with 1398, 1525, 1990, and 5541 images respectively, as marked in the ‘Main-Dynasty’ column of Table 3.

### 3.2. Research Models

Traditional machine learning methods and neural networks can all be used for image classification. However, traditional approaches like KNN, SVM, and random forest rely on feature selection for individual tasks, for instance, image intensity-related features for ceramic color recognition, and contour-related features for shape identification. Although machine learning approaches are more interpretable, without the labor-consuming work on parameters and features selection, KNN, SVM, and random forest with default parameters and flattened image as features can only obtain 44.88%, 26.51%, and 32.47% accuracy accordingly on the simplest color recognition task. On the other hand, deep neural networks are strong tools to extract complicated feature representation directly from large amounts of data; hence, we are interested in their ability for ceramic identification, especially for the much more difficult dating task.

**Figure 4:**
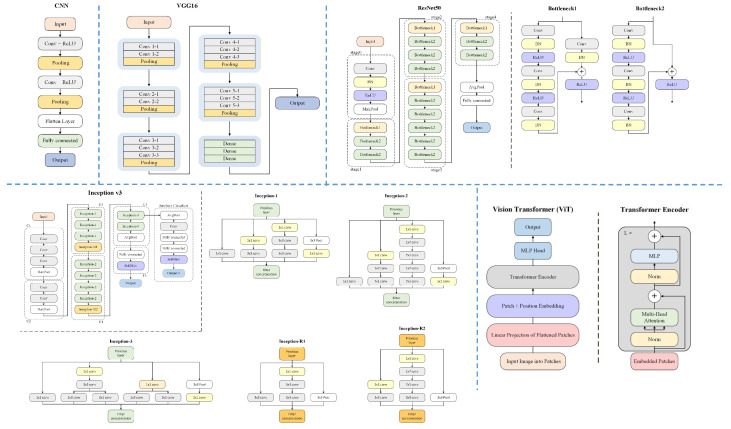
Network structures of CNN, VGG16, ResNet 50, Inception v3 and ViT.

To study the capacity of deep learning for early Chinese ceramic identification, 5 deep learning models from the simple to the most advanced in recent years, namely a simple 3-layer CNN, VGG16 (representing all VGG series), ResNet50, Inception-v3, and ViT are validated in two feature recognition tasks, and most importantly, the ceramic dating task. The network structures are demonstrated in Figure 4. CNN, VGG16, ResNet 50, and ViT share the same input image size of [224, 224, 3], while Inception-v3 uses [299, 299, 3].

### 3.3. Class-Imbalance Learning

Data imbalance is a severe problem in our work, especially for the shape recognition task and ceramic dating on a large scale, where the top 6 common shape types, i.e., Bowls, Plates, Bottles, Pots, Jugs, and Cups, make up nearly 81% of all collection, and the 4 main dynasty samples almost 86% of all dynasty dataset. Hence many classes have very limited data for feature extraction and are easily overwhelmed by the majority classes. In this work, a class-imbalance learning strategy is proposed to improve the robustness: (1) For models deeper than the 3-layer CNN, we pre-trained on ImageNet-1k and use fine-tuning to prevent overfitting. (2) A ceramic-image-specific data augmentation method is introduced to balance the training data.

Inspired by approaches like Random Erasing [26], a ceramic-image-specific augmentation is proposed to enforce important feature learning with randomly generated occlusion. For each class that has fewer samples compared with the biggest cluster, our augmentation strategy works as follows:

1. Randomly select a training image from the class to be extended.
2. Considering the collected images are of simple backgrounds of monochrome or gradient colors, pre-processing using Grab-Cut initialized with an inner rectangle is applied for ceramic segmentation.
3. Then, an occlusion rectangle is generated as follows: the center is randomly selected by the Gaussian distribution with a mean equal to the center of the foreground, and variance equal to the half distance of the foreground width and height. The rectangle width and height are randomly selected from a uniform distribution [0.05, *r*], where *r* is determined by the square root of the area ratio of foreground and image. Finally, the color of the rectangle is set to the median of channel intensities of the top part from the background.
4. Repeat processes 1–3 until the target class has an equal number of training images as the largest cluster.

**Figure 5:**
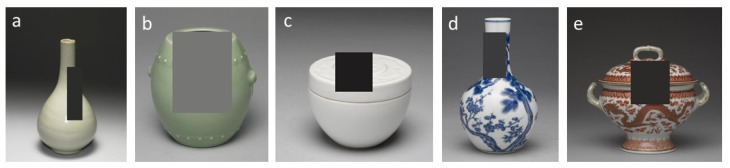
Augmented images. (**a**) Celadon Gall-Bladder Shaped Vase, (**b**) Celadon Glazed Drum-Shaped Jar with Animal-Head Handles and Concealed Ring Decorations, (**c**) White-Glazed Seed-Pod Shaped Box, (**d**) Blue-and-White Vase with the Three Friends of Winter Motifs (Underglaze), (**e**) Red-Enameled Gilt Jar with Cover Decorated with Double Dragons Chasing a Pearl Motifs (Overglaze).

Figure 5 illuminates some of our augmented images. Compared with oversampling, our data augmentation strategy generates random occlusions on ceramic boundaries or motif patterns and hence enforce the neural networks to remember the most relevant features.

**Figure 6:**
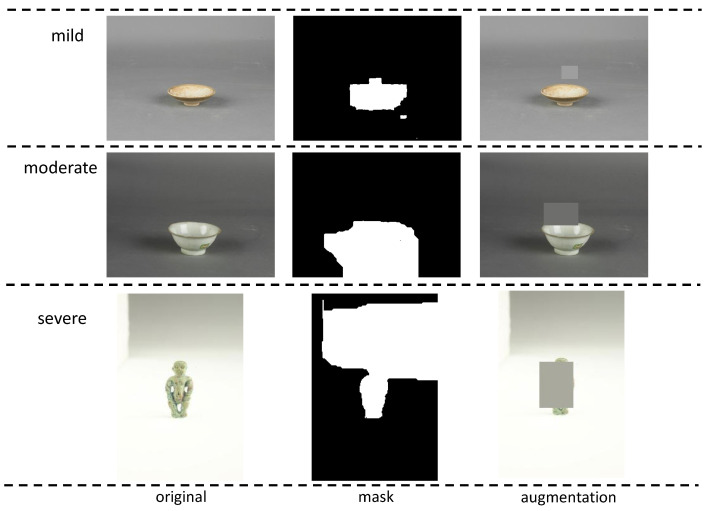
Failure cases of grab cut with low-quality images. From top to the bottom, we present mild, moderate, and severe segmentation failure cases on Celadon plate, Celadon bowl and Green-glazed figure, where the over-segmentation can lead to ineffective image augmentation.

Note that our GrabCut segmentation is based on the assumption that the collected images are of simple monochrome/gradient backgrounds, but a small number of images are of relatively low quality due to shadows, reflections, and image compression, which can cause foreground over-segmentation and lead to ineffective augmentation. As demonstrated in Figure 6, in the mild and moderate over-segmentation cases, the generated occlusion does not necessarily cover the main body of the ceramics, thus contributing little to data augmentation. Meanwhile, severe over-segmentation can produce overly large masks on small objects, leading to the loss of useful information.

### 3.4. Evaluation Criteria

Precision, recall, and F1 score as seen in Equation (1) are used as criteria for the recognition abilities of deep neural networks. All three criteria are based on True Positive (TP), False Positive (FP), False Negative (FN), and True Negative (TN): Precision represents the percentage of all samples that are correctly predicted to be positive, Recall represents the percentage of positive samples that are predicted to be positive, and F1 score is a metric that combines precision and recall as the harmonic mean.

(1) $$Accuracy=\frac{\sum_{i=1}^{m}I\left(y_{i}={\hat{y}}_{i}\right)}{m};Precision=\frac{\sum_{i=1}^{m}TP_{i}}{\sum_{i=1}^{m}\left(TP_{i}+FP_{i}\right)};Recall=\frac{\sum_{i=1}^{m}TP_{i}}{\sum_{i=1}^{m}\left(TP_{i}+FN_{i}\right)}$$

Given the severe class imbalance problem in our work, both weighted and macro averages of Precision, Recall, and F1 score are used to evaluate the model’s performance. For multi-label classification tasks, the weighted average calculates the same metric for each label and finds the average weighted by the number of true instances for each label. On the other hand, the macro average of a metric calculates the same metric for each label and then finds the average without weighting, thereby treating all labels equally important. By using both, the weighted average focuses on the majority of classes while the macro average includes the effect of imbalanced data. Note that when the denominator is zero, the corresponding criterion is set to zero, and the weighted F1 score is not necessarily between precision and recall.

### 3.5. Feature Visualization

To understand if deep learning can truly extract well-acknowledged features, a visualization method called Grad-CAM is used to investigate how decisions are made with the encoded features by convolutional neural networks [27]. Grad-CAM generates heatmaps using feature maps and gradient information from the final layers of CNNs, highlighting the important regions in an input image that contribute to the model’s decision. This provides insights into how the model focuses on specific parts of an image for classification. The specific steps are as follows:

Firstly, compute the gradient of the feature map $A^{k}$ of a convolutional layer (usually the last layer) with respect to the score of class *c*, denoted as $\frac{\partial y^{c}}{\partial A^{k}}$, where $\partial y^{c}$ represents the score for class *c*.

Let *Z* represent the total number of elements in the feature map, the weights $\alpha_{k}^{c}$ for each feature map $A^{k}$ is obtained by global average pooling over its width, height, and channel dimensions as follows,

(2) $$\alpha_{k}^{c}=\frac{1}{Z}\sum_{i}\sum_{j}\frac{\partial y^{c}}{\partial A_{ij}^{k}}$$

Finally, class activation mappings are generated by a weighted combination of feature maps and the application of the ReLU activation function. This process highlights the regions that are most critical for class *c* prediction.

(3) $$L_{Grad-CAM}^{c}=ReLU\left(\sum_{k}\alpha_{k}^{c}A^{k}\right)$$

## 4. Experiment Results

In this section, we compared the ability of five state-of-the-art neural networks on early Chinese ceramics’ feature recognition and dating. Although we strived to only collect representative images of ancient Chinese ceramics for the dataset, a subset of the images still exhibits a high degree of similarity due to specific popular ceramic wares often being produced in sets. For a fair comparison, we therefore first filtered all images by the ceramics’ names and their production time, arriving at 9087 unique wares out of the original datasets consisting of 12,253 images. We then partitioned these 9087 unique pieces of ceramic ware into training, validation, and test sets at a ratio of 6:2:2. Finally, the previously filtered images were added to the dataset containing the image sharing their name and production period, yielding the final task-specific training, validation, and test datasets.

For each task, we compared the performance of five neural networks in four training configurations: (1) a vanilla training strategy (Vanilla Models) using Cross Entropy Loss, the class-imbalance focused methods (2) Weighted Cross Entropy Loss (Weighted Loss), where the weights are set to the reciprocal of the sample size for each category, and (3) Focal Loss with γ=2. All three are compared to (4) our class-imbalance learning strategy (Optimized Models) with cross entropy loss. Note that VGG16, ResNet50, Inception, and ViT are pre-trained by ImageNet-1k and refined by our ceramic-image-specified data augmentation, while CNN uses our data augmentation only, which directly demonstrates the efficiency of our data augmentation method by itself.

All models share the same training hyperparameters, including epoch = 100, batch size = 32, SGD optimizer with learning rate = 0.01 and momentum = 0.9, and StepLR scheduler with step size = 20 and γ=0.5. The random seeds for all experiments are fixed to 123. The average number of epochs until convergence for all models and tasks is 76.06. Given the slightly different digitization standards of the collected images, the same basic data argumentation strategy is used in all tasks to improve the model’s robustness against different photograph angles, illumination conditions, and reflections. The augmentation consists of random horizontal and vertical flips, random rotation, and affine transformation, as well as random color adjustment of brightness, contrast, saturation, and hue.

All models are trained on the same training set, where models with the best accuracy on the validation set are saved and compared on the same testing set in the corresponding tasks. Models with the highest macro F1 score are considered the best, and the confusion matrix and classification Precision, Recall, and F1 scores of each label are present for further analysis.

### 4.1. Ceramic Feature Recognition Task

**Figure 7:**
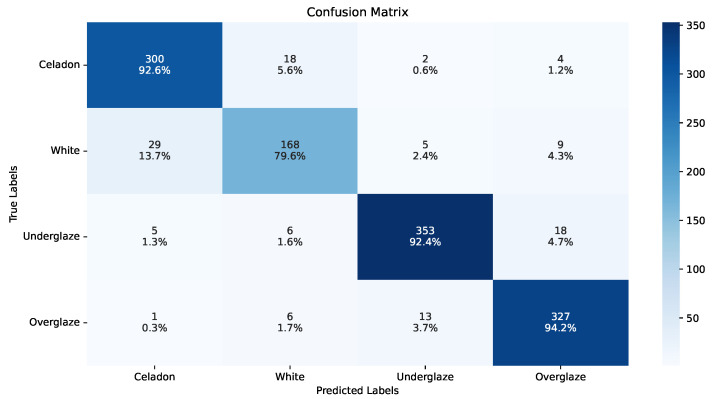
Confusion matrix of the best model on color identification. Each grid presents the number and percentage of samples with label corresponding to the y-axis being recognized as the class along the y-axis.

Ceramic feature recognition experiments are tested on the two aforementioned glaze color and shape datasets. For color identification, the samples of the four labels, celadon, white, underglaze, and overglaze, are relatively balanced. Results in Table 4 show that all models with different training strategies perform well on this task, indicating that color is a very distinguishable visual characteristic. While Weighted Loss or Focal Loss can improve the model performance, our training strategy has achieved better results on all models. ViT outperforms the others with the best 90.78% weighted F1 score, and a similar 89.87% macro F1 score. The similar weighted and macro scores indicate the class-imbalance is not severe in this task. Figure 7 and Table 5 are the optimized ViT’s confusion matrix and model performance on each label, demonstrating that all colors can be recognized with very high accuracy, with the exception of some classification errors between white glaze and celadon.

For shape recognition, the shape type dataset includes 15 highly imbalanced classes with 9 of them having fewer than 500 images. As shown in Table 6, all vanilla models perform much worse compared to color identification, while the macro F1 score is at least 10% less than the weighted F1 score. By using the proposed optimizations, all models’ performances are significantly improved. While Wighted Loss and Focal Loss improve over base models in some cases, our method outperforms both consistently. The optimized CNN model using only our ceramic-image-specific data augmentation improves the weighted F1 score by 7.14% from 55.04% to 62.18% and the macro F1 score by 10.83% from 39.45% to 50.82% compared with the vanilla model, showing the effectiveness of our data augmentation method. Deeper models like VGG16, ResNet50, and ViT have similar performances on this task with our optimization strategy, while ViT has the optimal weighted and macro F1 scores. In the best performing ViT, our optimization improves the weighted F1 score by 36.36% to 79.49%, and the macro F1 score by 43.56% to 72.80%.

**Figure 8:**
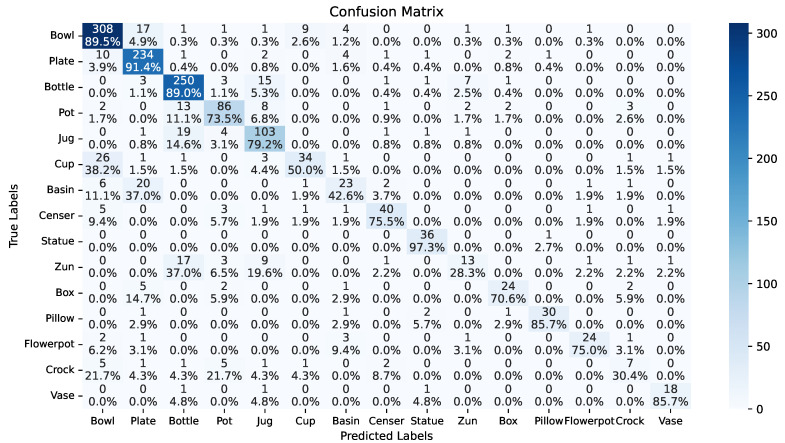
Confusion matrix of the best model on shape identification. Each grid presents the number and percentage of samplers with label corresponding to the y-axis being recognized as the class along the y-axis.

Figure 8 and Table 7 demonstrate the optimized ViT’s confusion matrix, precision, recall and F1 score on each label. Results show that shapes with sufficient samples can be recognized with very high accuracy. The identification precision on Bowl, Plate and Bottle with over 1600 samples for example is over 85% F1 score, while shapes like Basin, Zun, and Crock are of low accuracy under 50% F1 score. Unique shapes like Pillow (Figure 3) and Statue (see Section 5.3 for an example) are still easy to recognize with 89.55% and 91.14% F1 score despite only 190 and 293 samples, while 38.2% of Cups are mistaken as similar shaped Bowls, indicating the limitation of shape recognition using only one 2D image.

### 4.2. Ceramic Dating Task

To answer the main question of our study, that if deep learning can be used for ceramic dating with only one optical image, two experiments are conducted on an All-dynasty dataset and its subset, a Main-Dynasty dataset.

The All-Dynasty dataset covers ceramics from a large time span from Neolithic to Modern, including almost all 15 periods/dynasties except the Qin Dynasty (only 1 sample). Table 8 presents the performances of 5 neural networks on this dataset. Although our optimized models improve over vanilla models, weighted loss and focal loss results in all tested models, the results show that ceramic dating is the most difficult task in this study. The best weighted F1 score is obtained by VGG16 at 82.45%, but the corresponding imbalance-sensitive macro F1 score is only 46.11%, showing a strong focus on classes with larger amounts of samples. Although very low in general, our optimized augmentation strategy manages to improve macro F1 scores by at least 20.11%. The optimal macro F1 score is achieved by ViT with 52.25% while having the second highest weighted F1 score of 82.09%.

**Figure 9:**
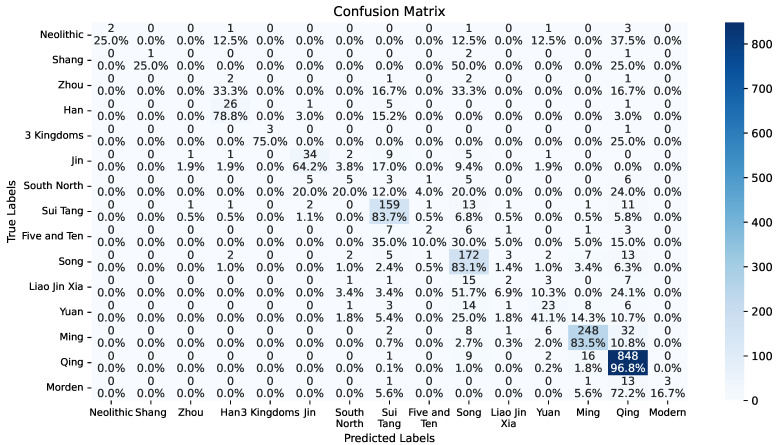
Confusion matrix of the best model on All-Dynasty dating. Each grid presents the number and percentage of samplers with label corresponding to the y-axis being recognized as the class along the y-axis. Note that Southern Northern is short for Southern and Northern Dynasties, Five and Ten is short for Five Dynasties and Ten Kingdoms. Liao Jin Xia is short for Liao, Jin, and Western Xia.

Figure 9 and Table 9 show the classification accuracy details of the best-performing model ViT with our class-imbalance learning on each dynasty or period. As can be seen, the model performances on small classes like Neolithic, Shang, Zhou, Five dynasties and Ten Kingdoms, Liao and Jin and Western Xia and modern Periods are dramatically low, showing the influence of these highly imbalanced classes.

**Figure 10:**
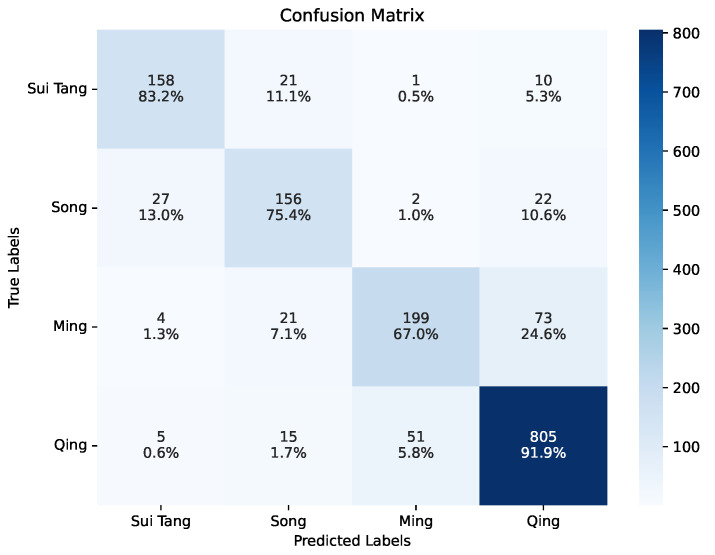
Confusion matrix of the best model on Main-Dynasty dating. Each grid presents the number and percentage of samplers with label corresponding to the y-axis being recognized as the class along the y-axis.

Considering the poor performance caused by an insufficient amount of learning data, a second experiment is conducted on the Main-Dynasty dataset, with the four main dynasty samples covering 86% of the All-Dynasty dataset. As shown in Table 10, focusing on these dynasties improves the performances of all models by a large margin and still greatly benefits from class-imbalance learning, compared with the models trained with various Loss functions. The best performance is obtained by ViT with 83.74% weighted F1 score and most importantly, 79.77% macro F1 score. VGG16 achieves the second best performance with 80.96% and 75.77% weighted and macro F1 scores. The confusion matrix and evaluation metrics of the optimized ViT on each label are presented in Figure 10 and Table 11. The results show that the Ming and Qing ceramics are easily misclassified as each other, where 24.6% Ming ceramics are predicted as Qing dynasty productions. Moreover, although the class number is reduced to only 4, the identification performance on the Ming and Qing dynasties is decreased, indicating that the samples from other dynasties can still contribute to the feature learning and overfitting prevention, thereby leading to better prediction performance on the majority classes.

## 5. Discussion

Our results show that visual features like glaze color are relatively easy to recognize by most of the selected deep learning models, with up to 89.87% macro F1 scores, while the optimal shape type recognition is 72.8% macro F1 score with our class-imbalance learning strategy. Deep learning models demonstrate strong ability in visual feature learning with sufficient data and on unique type shapes like Statue and Pillow, but also show limitations on distinguishing categories with high inter-class similarity using 2D images only.

Ceramic dating appears to be a much harder task due to the severe class-imbalance, exhibiting significant performance inconsistency measured by weighted and macro metrics. But the prediction performances on the long, peaceful, and prosperous dynasties like Han, Sui Tang, Song, Ming, and Qing are over 74.95%, indicating that early Chinese ceramics do have relatively distinguishable characteristics related to the production period. The further comparison on the Main-Dynasty dataset reflects that deep learning models are able to recognize the special visual features formed within the development over time.

The ancient Chinese ceramic dating results demonstrate that ceramics are prone to misclassification as products of neighboring dynasties. This observation implies the cultural inheritance between continuous historical periods. Hence, further analysis is conducted to investigate this phenomenon.

### 5.1. Impact of Cultural Inheritance

**Figure 11:**
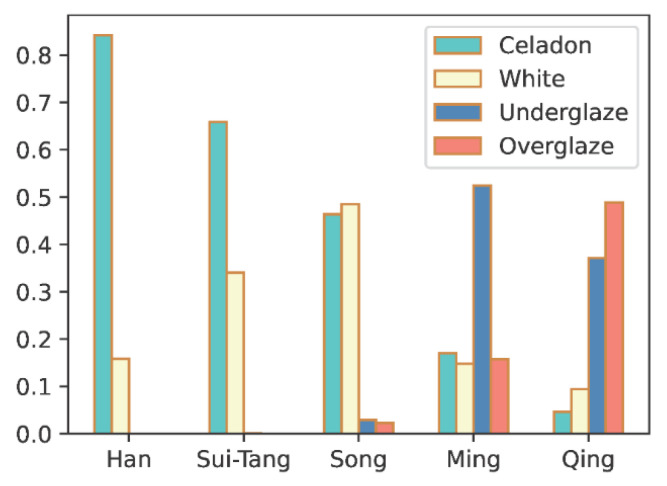
Histogram of celadon, white, underglaze, and overglaze ceramics pieces from Han, Sui Tang, Song, Ming, and Qin.

The development history of glaze color reveals that visual features can be shared in proximate periods. This fact can also be proven by the distribution of the 4 studied colors in our collected ceramics. Figure 11 shows the trends in distribution of celadon, white, underglaze, and overglaze ceramics pieces from Han, Sui Tang, Song, Ming, and Qin. As can be seen, celadon potteries were already widely used in the Han dynasty. The matured firing techniques further brought white-glazed pottery or porcelain in the following dynasties and eventually overtook the popularity of celadon in the Song dynasty. Underglaze like the representative Blue-and-White porcelain started from Song and peaked in Ming. The more complicated firing skill also created overglaze of various bright colors, and became the most popular ceramic wares in the Qing dynasty.

Figure 11 shows that although each historical period has its major colors, the features are shared in a much larger time span. This fact can consequently lead to reasonably inaccurate ceramic dating results. As demonstrated by the two confusion matrices for ceramic dating in Figure 9 and Figure 10, ceramic dating by deep learning is more likely to misclassify close dynasties. Especially in the Main-Dynasty dating task, 28 out of 34 misclassified Sui Tang ceramics are identified as from the following Song dynasty, 27 out of 51 misclassified Song ceramic wares are recognized as from the Sui Tang period, 73 out of 98 misclassified Ming wares are taken into Qing ceramics, and 51 out of 71 misclassified Qing ceramics are identified as from the previous Ming dynasty. The overall error rates in the neighboring periods are 65.63%, 56.86%, 95.92%, and 71.83% for the Sui Tang, Song, Ming, and Qing dynasties or periods, respectively.

Hence, one can conclude that cultural inheritance, like firing and glaze techniques cannot be simply periodized by the division of history into corresponding dynasties. Even though current neural networks can recognize time-relevant features with very high accuracy, relying solely on visual information can still lead to “reasonable” misclassifications in ceramic dating.

### 5.2. Temporal Distance Comparison

**Figure 12:**
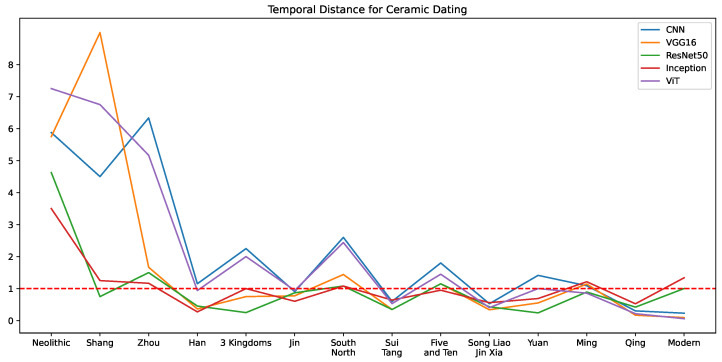
Temporal distance of the 5 optimized models’ predictions for All-Dynasty dating. Note that the Song, Liao, Jin, and Western Xia dynasties are categorized under the same temporal period in our study. The x-axis ticks represent dynasties or periods in the historical sequence. Temporal distance 1 (red dashed line) means the prediction lies in the neighboring dynasty or period.

A more rigorous quantitative analysis using temporal distance of the predicted dynasty or period is conducted to clarify the cultural heritage and artistic continuity in ancient Chinese ceramic production. In this subsection, we labeled the dynasties or periods by consecutive ordinal numbers according to their historical chronological sequence. Note that Song, Liao, Jin and Western Xia coexisted roughly within a shared historical period, these dynasties are clustered with one index in this temporal distance research. Hence, we labeled the dynasties or periods with index from 1 to 14, calculated the absolute difference of the predicted label to the real index, and demonstrated the prediction error of all 5 models at each dynasty or period in Figure 12. The result shows that other than the very ancient periods like Neolithic, Shang and Zhou which also have very few samples (37, 13, 33 images respectively), the average temporal prediction distances of all models are around 1. Comparing the results in Figure 9 and Figure 12, it can be concluded that cultural heritage maintains artistic continuity across successive periods, hence, the misclassified ancient ceramic artifacts without distinct visual transitions tend to be identified as belonging to temporally adjacent dynasties. Among all 5 networks, the classification error of ResNet50 on the main dynasties lower than 1, exhibiting the best robustness for chronological dating. ViT, which achieves the optimal macro F1 score, only performs similar to simple CNN before the Song, Liao, Jin and Western Xia periods and outperforms ResNet50 after the Ming dynasty.

Given this observation, we calculate the average temporal distance of all samples and at the average temporal distance of all classes, defined as weighted temporal distance and macro temporal distance as a performance measurement in Table 12. Moreover, a hierarchical evaluation where near-misses are partially credited is conducted. More specifically, predictions within the two adjacent periods are considered correct, and the corresponding weighted and macro temporal F1 scores of all models are compared with the original F1 scores from Table 8 in Table 12. As expected, the weighted and macro Temporal F1 scores of all models are higher than the original F1 scores, especially the macro F1 scores. VGG16 achieves the highest weighted temporal F1 score of 91.52%, while ViT performs best at the macro temporal F1 score of 76.16% and second best at both weighted F1 scores. Meanwhile, ViT obtained the lowest weighted temporal distance 0.0594, while ResNet50 has the optimal macro temporal distance 1.0009. The comparison across multiple criteria shows that ViT and VGG16 have better capacity to recognize the true production period of most samples, but they should be improved in learning feature continuity across historical periods.

### 5.3. Model Interpretability

**Figure 13:**
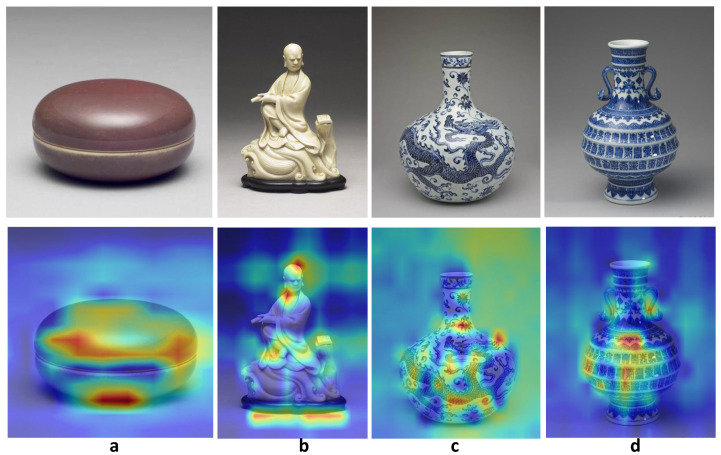
Examples of class activation mapping using the best performed ResNet50 model on All-Dynasty dataset, showing the features that contribute to the dating decision. (**a**) Vine Bean Red-Glazed Small Box; (**b**) White Arhat Statue; (**c**) Blue-and-White Globular Vase with Dragon-and-Lotus Intertwined Motifs; (**d**) Blue-and-White Ruyi-Handled Zun Decorated with Hundred Shou Motifs. The top row shows original images, and bottom row shows activation maps generated by Grad-CAM, where color from cold to warm demonstrates the features’ contribution from low to high.

Given the success of ceramic color and most shape type classification, it is possible that deep learning could recognize commonly acknowledged visual features. In this subsection, we further investigate how this feature extraction works for ceramic dating. Using pre-training and our data augmentation method, ViT has outperformed all models across all tasks, achieving greatly improved macro F1 scores. Taking the optimized ViT in the All-Dynasty experiment for example, Grad-CAM is used to visualize the learned features by its influence on the final classification. Figure 13 presents some example images of ceramics from different dynasties and their activation heatmap of features according to the predicted class.

The results on special shapes like box (Figure 13a) and Statue (Figure 13b) suggest that the network trained on the dating dataset is still sensitive to visual features like shapes. Moreover, the heatmap of the round box shows higher activation along its round shape and the edge of the box lid, indicating that the vine bean red-glaze is a distinct visual feature for ceramic dating, as this glaze was created in the Qing dynasty. The heatmap of the Arhat statue highlights the face and the hem of a monk’s robe, implying that the neural networks could recognize decoration techniques for human figures and their attire as important indicators for the production time. Meanwhile, the comparison of the heatmaps on two Blue-and-White porcelains from two different dynasties (the vase in Figure 13c is from the Ming dynasty, and the Zun in Figure 13d is from the Qing dynasty) illuminates that the unique shape and motif features are used in prediction, highlighting their importance in ceramic dating. In the Qing and Ming Dynasties, Blue-and-White porcelain developed into various shapes and rich motif pattern styles, which became important features for ceramic dating. Our result shows that the neural network could recognize the globular vase shape from the Ming Dynasty, and the special Ruyi-Handled Zun shape with its complicated angular surface and dense longevity character motifs, thus indicating its Qing Dynasty origin due to a sophisticated, highly complex firing technique.

These findings reveal the potential of deep learning in detailed feature or unlabeled feature recognition, and also provide ideas and directions for further quantitative research on the evolution of Chinese porcelain styles and patterns across different dynasties. Although these Grad-CAM interpretations are only speculative based on gradient activation, they give insight into possible reasons for the model’s ability to distinguish production periods not only based on shape, but also on patterns. However, deep learning outcomes do not equate to professional historical interpretations, and domain knowledge is still required to identify the distinct visual features for dynastic dating. Moreover, for visualization techniques like Grad-CAM, the high compression of feature maps also makes it difficult to accurately locate visual clues.

## 6. Conclusions

In this paper, we validate the feasibility of neural networks to identify early Chinese ceramics with a single 2D image. The performances of five state-of-the-art deep learning models are studied on ceramic datasets for both visual feature recognition and early Chinese ceramic dating tasks.

We conclude our work with answers to the question proposed in the beginning, that deep learning can identify early Chinese ceramics using only one 2D image, and identify the production period of most ceramic wares with high accuracy on the important periods of Chinese ceramic development history. Class-imbalance is a severe problem for tasks of diversified shape recognition or All-Dynasty dating, with proper optimization like our class-imbalance learning strategy, deep learning is still able to reach a high accuracy with sufficient learning data.

Further analysis reveals that cultural inheritance of ceramic production keeps an artistic continuity on relatively close dynasties, which leads to a reasonable near-miss by classifying the ambiguous ceramics into adjacent periods. Meanwhile, our model interpretation work shows that neural networks have the potential to capture visual features beyond color and shape, and could be a tool to connect them to the corresponding production and decoration styles or skills. Although domain knowledge is still required to identify the correlation between visual features and ceramic dating, model visualization techniques can definitely help popularize science about early Chinese ceramics in museums.

Our work demonstrates the potential of deep learning in ancient Chinese ceramic feature identification and dating, but it should be used with caution compared with professional historical interpretations. Current deep neural networks also show limitations in recognizing ambiguous shapes and production periods, especially with highly compressed images. In future work, we will collaborate with domain experts to develop a fine-grained classification system for time-relevant visual features, such as glaze colors and shape variants, and to examine the evolution of these visual clues across the history of ancient Chinese ceramics.

## Figures and Tables

**Table 1:** An overview of the glaze color dataset.

Glaze Color Dataset				
**Color**	**Celadon**	**White**	**Underglaze**	**Overglaze**
Number	2464	1733	2436	2284

**Table 2:** An overview of the shape type dataset.

Shape Type Dataset							
Main-Shape	Num	Sub-Shape (English/Chinese)	Num	Main-Shape	Num	Sub-Shape (English/Chinese)	Num
Bowl	2861	Bowl (碗)	2792	Censer	338	Censer (炉)	338
		Alms Bowl (钵)	69	Statue	293	Figure (俑)	232
Plate	1948	Plate (盘)	1698			Statue (像)	61
		Saucer (碟)	250	Zun	276	Zun (尊)	276
Bottle	1653	Bottle (瓶)	1653	Box	201	Box (盒)	201
Pot	934	Pot (罐)	934	Pillow	190	Pillow (枕)	190
Jug	819	Jug (壶)	819	Flowerpot	170	Flowerpot (花盆)	155
Cup	523	Cup (杯)	462			Flowerpot Cover (奁)	15
		Handless Cup (盅)	42	Crock	121	Crock (缸)	121
		Tea Bowl (盏)	19	Vase	113	Gu-shape-Vase (瓷觚)	97
Basin	346	Small Basin (洗)	320			Receptacle (花插)	16
		Basin (盆)	26	Others			

**Table 3:** An overview of two early Chinese ceramic dating datasets: the all-dynasty dataset and the main-dynasty dataset.

Dynasty Datasets		
**Main-Dynasty**	**All Dynasty**	**Num**
	Neolithic (6000 BC–2000 BC)	37
	Shang (ca. 2146 BC–1029 BC)	13
	Zhou (ca. 1029 BC–221 BC)	33
	Qin (ca. 221 BC–207 BC)	1
	Han (206 BC–220)	232
	Three kingdoms (220–280)	41
	Jin (265–420)	431
	Southern and Northern Dynasties (420–589)	182
✓	Sui Tang (581–907)	1398
	Five Dynasties and Ten Kingdoms Period (905–960)	123
	Liao and Jin and Western Xia (916–1234)	208
✓	Song (960–1279)	1525
	Yuan (1271–1368)	369
✓	Ming (1368–1644)	1990
✓	Qing (1616–1911)	5541
	Modern (1912–now)	100

The main-dynasty dataset is highlighted in gray.

**Table 4:** Comparison of the model’s overall performance on color identification. The criterion consists of weighted/macro F1 score (%).

	Vanilla Models	with Weighted Loss	with Focal Loss	Optimized Models
CNN	83.10/81.81	82.53/81.39	81.65/80.41	83.30/82.13
VGG16	83.29/82.03	84.01/82.89	83.99/82.76	84.84/83.75
ResNet50	83.90/82.59	82.90/81.56	82.23/81.08	83.61/82.17
Inception	55.67/50.58	49.36/47.69	51.02/46.65	89.21/88.11
ViT	82.21/80.79	82.70/81.50	81.44/80.35	90.78/89.87

Models with the best weighted/macro F1 scores are highlighted in blue.

**Table 5:** Best performance on color identification (%).

	Precision	Recall	F1 Score
Celadon	89.55	92.59	91.05
White	84.85	79.62	82.15
Underglaze	94.64	92.41	93.51
Overglaze	91.34	94.24	92.77

**Table 6:** Comparison of the model’s overall performance on shape recognition. The criterion consists of weighted/macro F1 score (%).

	Vanilla Models	with Weighted Loss	with Focal Loss	Optimized Models
CNN	55.04/39.45	50.83/39.67	54.92/39.90	62.18/50.28
VGG16	64.73/53.23	62.31/53.80	66.36/55.69	78.61/72.10
ResNet50	57.39/43.14	39.69/32.60	59.45/47.81	77.26/72.32
Inception	31.63/13.80	19.90/15.13	28.77/12.26	74.08/70.49
ViT	43.13/29.24	34.28/27.07	42.87/27.91	79.49/72.80

Models with the best weighted/macro F1 scores are highlighted in blue.

**Table 7:** Best performance on shape identification (%).

	Precision	Recall	F1 Score
Bowl	84.62	89.53	87.01
Plate	82.39	91.41	86.67
Bottle	82.24	88.97	85.47
Pot	80.37	73.50	76.79
Jug	71.53	79.23	75.18
Cup	73.91	50.00	59.65
Basin	60.53	42.59	50.00
Censer	81.63	75.47	78.43
Statue	85.71	97.30	91.14
Zun	52 00	28.26	36.62
Box	77.42	70.59	73.85
Pillow	93.75	85.71	89.55
Flowerpot	85.71	75.00	80.00
Crock	43.75	30.43	35.90
Vase	85.71	85.71	85.71

**Table 8:** Comparison of the model’s overall performance on All-Dynasty dating. The criterion consists of weighted/macro F1 Score (%).

	Vanilla Models	with Weighted Loss	with Focal Loss	Optimized Models
CNN	58.79/20.46	50.32/24.38	58.69/20.86	76.43/46.55
VGG16	59.35/19.32	36.63/6.30	60.67/21.50	82.45/46.11
ResNet50	61.14/21.18	40.92/18.87	60.28/21.99	75.96/48.98
Inception	35.51/7.67	8.48/3.51	32.75/6.87	64.68/39.10
ViT	63.09/32.14	49.0/25.93	63.45/32.59	82.09/52.25

Models with the best weighted/macro F1 scores are highlighted in blue.

**Table 9:** Best performance on All-Dynasty dating (%).

	Precision	Recall	F1 Score
Neolithic	100	25.00	40.00
Shang	100	25.00	40.00
Zhou	0.00	0.00	0.00
Han	78.79	78.79	78.79
3 Kingdoms	100	75.00	85.71
Jin	80.95	64.15	71.58
South North	45.45	20.00	27.78
Sui Tang	80.71	83.68	82.17
Five and Ten	40.00	10.00	16.00
Song	68.25	83.09	74.95
Liao Jin Xia	22.22	6.90	10.53
Yuan	60.53	41.07	48.94
Ming	87.94	83.50	85.66
Qing	89.64	96.80	93.08
Modern	100	16.67	28.57

**Table 10:** Comparison of the model’s overall performance on Main Dynasty dating. The criterion consists of weighted/macro F1 Score (%).

	Vanilla Models	with Weighted Loss	with Focal Loss	Optimized Models
CNN	68.07/58.59	69.74/63.67	69.90/61.21	73.80/66.76
VGG16	69.71/60.63	70.91/63.32	67.98/58.63	80.96/75.77
ResNet50	73.28/65.28	65.05/59.79	69.84/61.71	72.63/70.09
Inception	46.99/30.52	25.65/29.76	45.51/30.84	76.59/72.93
ViT	72.86/66.29	66.54/62.0	71.57/64.82	83.74/79.77

Models with the best weighted/macro F1 scores are highlighted in blue.

**Table 11:** Best performance on Main-Dynasty dating (%).

	Precision	Recall	F1 Score
Sui Tang	81.44	83.16	82.29
Song	73.24	75.36	74.29
Ming	78.66	67.00	72.36
Qing	88.46	91.89	90.15

**Table 12:** Comparison of models’ performance on All-Dynasty dating task, using weighted/macro original F1 scores (%), temporal F1 score (%), and temporal distance.

	Original F1	Temporal F1	Temporal Distance
CNN	76.43/46.55	86.34/72.99	0.2317/2.1129
VGG16	82.45/46.11	91.52/74.75	0.0947/1.6812
ResNet50	75.96/48.98	83.48/69.72	1.0023/1.0009
Inception	64.68/39.10	77.27/63.09	1.3379/1.0566
ViT	82.09/52.25	90.40/76.16	0.0594/2.1435

Models with the best weighted/macro F1 scores are highlighted in blue.

## Data Availability

The dataset supporting the findings of this study is available from the corresponding author upon reasonable request.
